# Pathologic Complete Response of HER-2 Neu-Positive Invasive Ductal Carcinoma and Ductal Carcinoma *In Situ* following Neoadjuvant Chemotherapy plus Trastuzumab: A Case Report and Review of Literature

**DOI:** 10.1155/2012/454273

**Published:** 2012-01-19

**Authors:** Sommer R. Gunia, Mita S. Patel, Eleftherios P. Mamounas

**Affiliations:** ^1^Department of Surgery, Affinity Medical Center, Massillon, OH 44646, USA; ^2^Department of Surgery, Akron General Medical Center, Akron, OH 44307, USA; ^3^Cancer Center, Aultman Health Foundation, Canton, OH 44710, USA; ^4^Northeastern Ohio Universities College of Medicine, Rootstown, OH 44272, USA

## Abstract

Pathologic complete response (pCR) after NC has been consistently associated with improved outcomes. Residual DCIS after NC does not portray worse prognosis compared to complete eradication of all disease but has clinical implications regarding surgical management. We report a case of pCR of DCIS associated with invasive carcinoma in an HER-2 + tumor after NC plus trastuzumab despite persistence of malignant-appearing microcalcifications mammographically. A 41-year-old Caucasian female presented with a 4 × 4 cm mass in the right breast and a 2.5 cm right axillary node. Mammogram showed a 2.5 cm mass and a 12 cm area of linear pleomorphic, suspicious calcifications in the upper part of the breast. Core biopsy revealed invasive ductal carcinoma and DCIS associated with calcifications (ER 85%, PR 6%, Her2neu 3+ by IHC). Axillary node FNA was positive for malignancy. The patient received doxorubicin/cyclophosphamide (AC) → paclitaxel plus T with complete clinical and radiologic response but no significant change in the microcalcifications. Final pathology showed no residual invasive carcinoma or DCIS despite the presence of numerous ducts with microcalcifications. Documented eradication of DCIS has not been reported following NC when malignant-appearing calcifications persist and this observation may have important clinical implications regarding surgical management.

## 1. Case Report

A 41-year-old female presented with a three-week history of palpable mass of the right breast and a three-day history of a palpable lump in the right axilla. Her past medical history was unremarkable except for history of thyroid insufficiency (on thyroid replacement) and a benign cyst removal from the left breast at the age of 14. She had menarche at the age of 13 and has one child (delivered at the age of 31). She is currently premenopausal and on birth control pills. Her family history is remarkable for breast cancer in two paternal cousins (diagnosed in their 40's), and for prostate cancer in her father (died at the age of 65).

**Figure 1:**
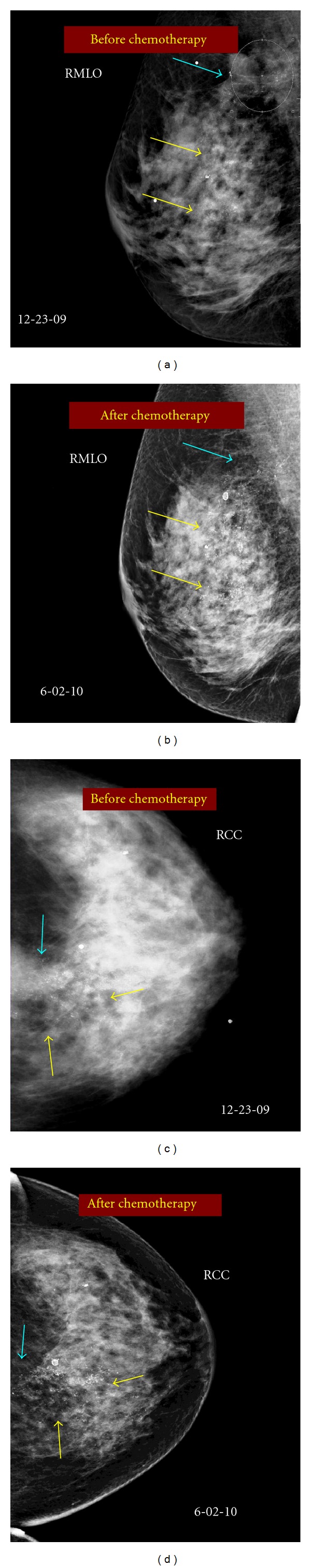
Comparison of mammographic studies before and after neoadjuvant chemotherapy. (a) Right MLO view prior to neoadjuvant chemotherapy. The blue arrow indicates the mass with surrounding microcalcifications. The yellow arrows indicate additional malignant-appearing microcalcifications occupying the upper part of the right breast. (b) Right MLO view after neoadjuvant chemotherapy. The blue arrow shows resolution of the mass but persistence of surrounding microcalcifications. The yellow arrows show persistence of the additional, extensive microcalcifications. (c) Right CC view prior to neoadjuvant chemotherapy. The yellow arrows indicate the areas of microcalcifications. The blue arrow indicates the breast mass. (d) Right CC view after neoadjuvant chemotherapy. The yellow arrows indicate the areas of persistent microcalcifications. The blue arrow shows complete resolution of the breast mass.

**Figure 2:**
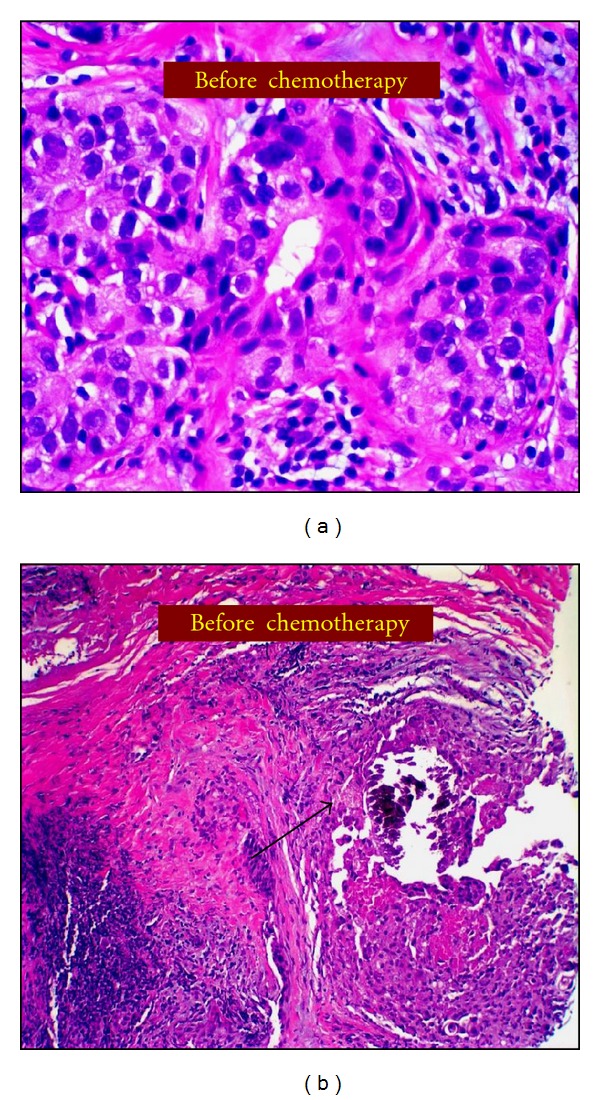
Pathologic findings from core needle biopsy before neoadjuvant chemotherapy. (a) H & E stain showing high-grade invasive carcinoma. (b) H & E stain demonstrating high-grade ductal carcinoma *in situ* associated with microcalcification (black arrow).

**Figure 3:**
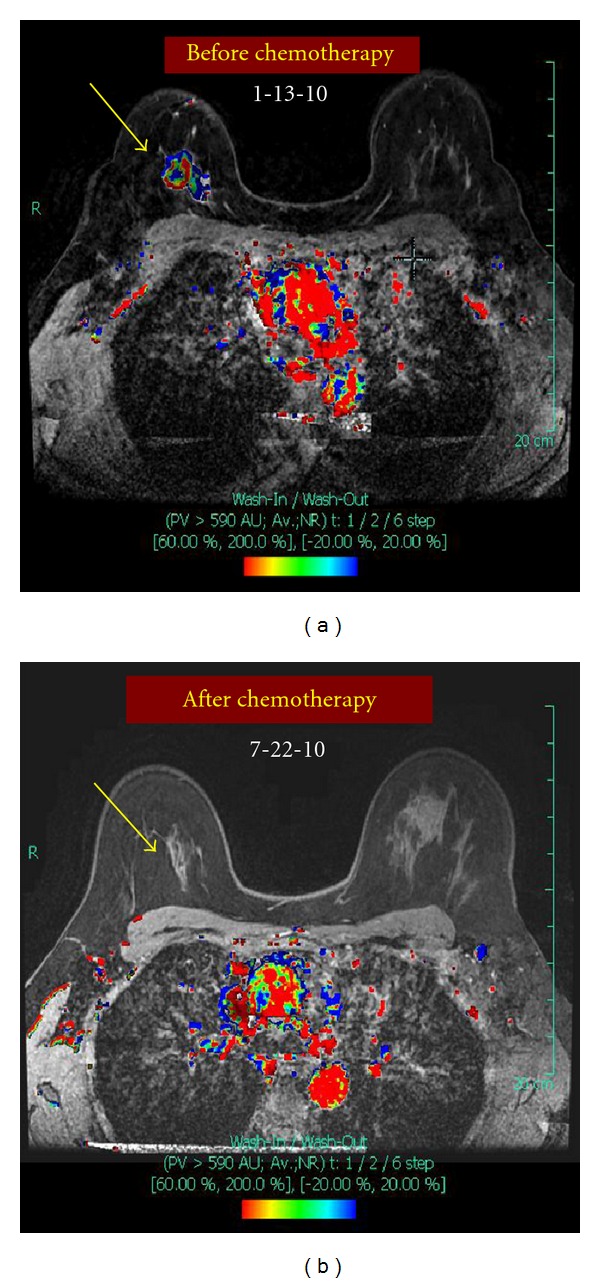
Comparison of bilateral breast MRI studies performed before and after neoadjuvant chemotherapy. (a) Doppler flow MRI before neoadjuvant chemotherapy. Yellow arrow points to the 2.7 cm irregular mass in the right breast. (b) Doppler flow MRI after neoadjuvant chemotherapy shows complete radiologic response. Yellow arrow points to area where the mass was located prior to treatment.

**Figure 4:**
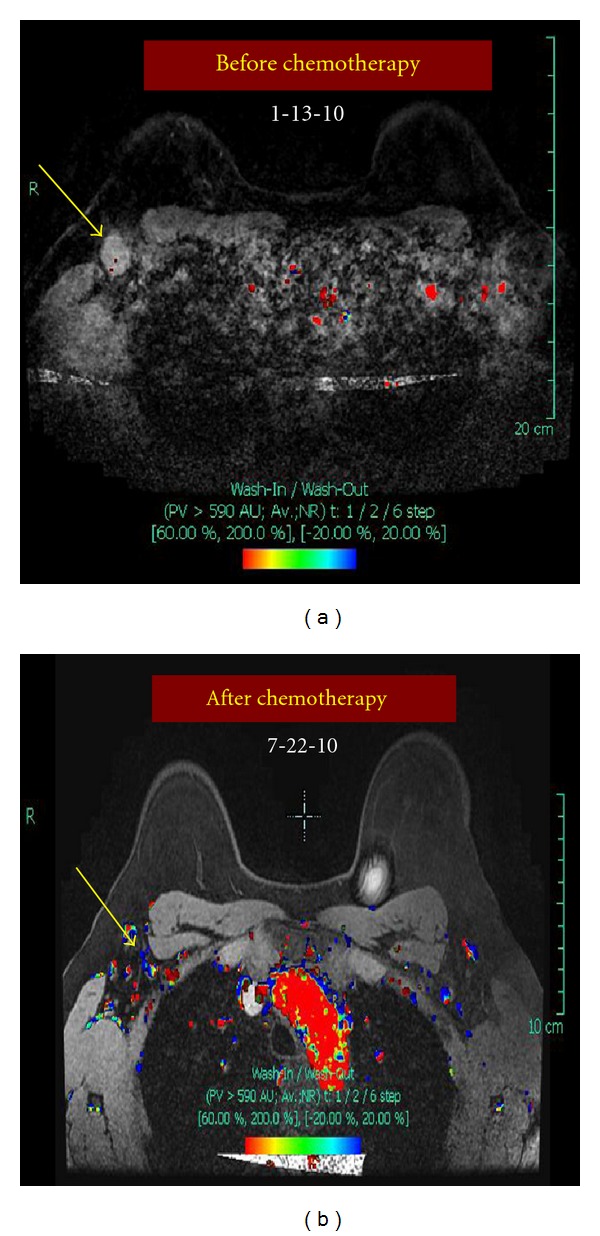
Comparison of bilateral MRI of the right axilla performed before and after neoadjuvant chemotherapy. (a) Doppler flow MRI before neoadjuvant chemotherapy. Yellow arrow points to a 2.6 cm pathologically enlarged right axillary lymph node. (b) Doppler flow MRI after neoadjuvant chemotherapy shows complete radiologic response. The yellow arrow points to the normal-appearing right axillary lymph node.

On examination, there was a 4 × 4 cm mass in the upper part of the right breast not fixed to underlying pectoralis muscle or overlying skin. There was also an enlarged right axillary node measuring approximately 2.5 cm in diameter, which was not fixed to surrounding structures. Digital mammography revealed a new 2.5 cm irregular solid mass at the 12 o'clock position of the right breast, which was highly suggestive of malignancy, and a 12 cm area of linear pleomorphic calcifications occupying most of the upper part of the right breast (Figures 1(a) and 1(c)). A targeted right breast ultrasound demonstrated an irregular solid, hypoechoic mass measuring 2.5 cm in diameter, and ultrasound of the right axilla confirmed the presence of a pathologically enlarged lymph node measuring 1.7 cm in diameter. The patient underwent an ultrasound-guided core biopsy of the right breast mass and an FNA of the axillary node. The core biopsy demonstrated invasive, moderately to poorly differentiated ductal adenocarcinoma, extensively involving both core biopsy fragments (Figure 2(a)). In addition, grade 3 DCIS, solid and clinging types, with comedonecrosis, and focal associated microcalcifications were seen (Figure 2(b)). Lymphovascular invasion was present. Immunohistochemical staining of the tumor showed strongly positive estrogen receptor (ER) at 85%, weakly positive progesterone receptor (PR) at 6%, and positive HER2neu overexpression (3+). The right axillary lymph node cytology confirmed the presence of metastatic adenocarcinoma consistent with breast origin. A bilateral breast MRI revealed a 2.7 cm irregular mass in the right breast (Figure 3(a)). In addition, multiple smaller foci of delayed washout type enhancement were also identified, predominantly involving the upper inner quadrant and the region central to the nipple in the posterior depth. These foci correlated with the pleomorphic calcifications seen on the comparison mammogram and were thought to most likely represent multifocal disease. In the right axilla, there was a 2.6 cm pathologically enlarged lymph node, likely metastatic (Figure 4(a)). Metastatic workup including CT scan of the chest/abdomen/pelvis and bone scan was negative.

The decision was made to proceed with neoadjuvant chemotherapy plus trastuzumab. The patient received four cycles of doxorubicin and cyclophosphamide (AC) followed by 12 cycles of weekly paclitaxel plus trastuzumab. After the first cycle of AC, there was a noticeable decrease in the size of the breast mass, and the axillary node was no longer palpable. The patient had complete clinical response following the four cycles of AC and maintained it while on paclitaxel plus trastuzumab. During this time, she was also referred to the Genetics Clinic for evaluation given her family history of breast and prostate cancer. She was found to be negative for BRCA-1 and BRCA-2 mutations including the BRAC analysis rearrangement test (BART).

Prior to surgery, a diagnostic mammogram of the right breast was obtained, showing no significant change in the diffuse pleomorphic calcifications, but complete resolution of the breast and axillary masses (Figures 1(b) and 1(d)). An MRI also showed complete radiologic response to treatment (Figures 3(b) and 4(b)). There was also no sonographic evidence of malignancy, and all axillary lymph nodes appeared benign.

**Figure 5:**
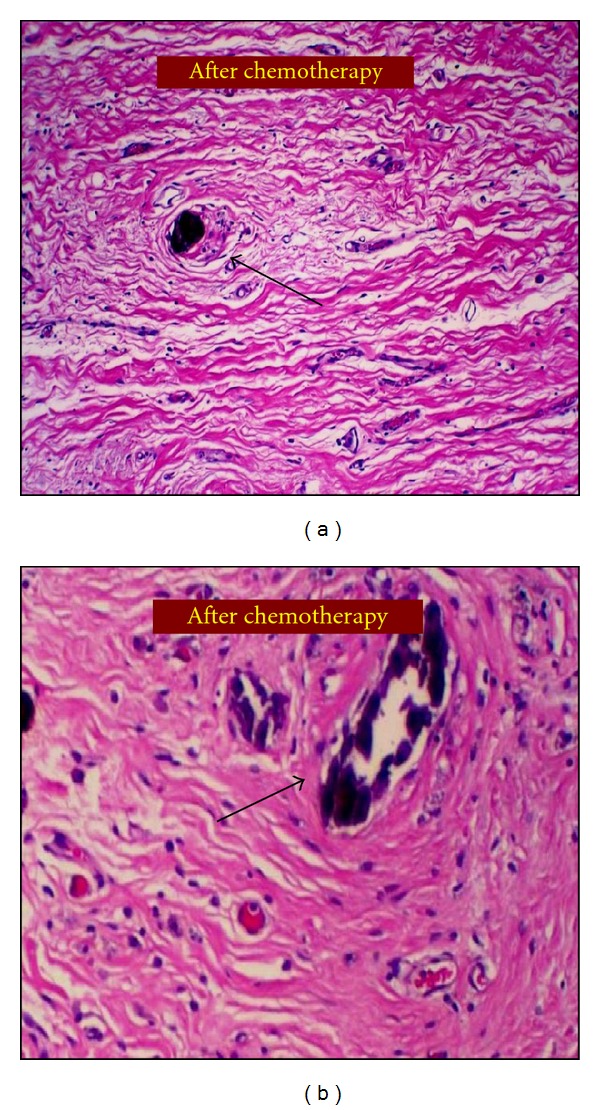
Pathologic findings from the surgical specimen obtained following neoadjuvant chemotherapy. Both pictures show no evidence of residual malignancy indicating a pathologic complete response. Black arrows point to benign appearing ducts with foamy macrophages, fibrous reactive tissue, and residual intraductal microcalcifications without evidence of DCIS.

Given the complete clinical response but persistence of extensive malignant-appearing microcalcifications present throughout the upper part of the breast, indicating extensive intraductal component, a total mastectomy was recommended. Because of her family and personal history indicating possible genetic predisposition (albeit not BRCA related), the patient opted for contralateral prophylactic mastectomy. Given the complete resolution of the right axillary node, she was entered into the ACOSOG Z071 trial evaluating sentinel node biopsy followed by completion axillary dissection in patients receiving neoadjuvant chemotherapy. Surgical treatment included bilateral skin-sparring mastectomy with right SNB and right completion axillary dissection. Bilateral submuscular tissue expanders were used for immediate reconstruction. Pathology demonstrated three negative sentinel lymph nodes and 17 negative nonsentinel lymph nodes. The biopsy cavity, 2.0 × 1.5 cm, showed no evidence of residual carcinoma or DCIS. The specimen contained numerous residual microcalcifications but no malignancy (Figures 5(a) and 5(b)). The patient was considered to have achieved a pathologic complete response (pCR).

The patient did well postoperatively although she had to undergo minor revision of the right mastectomy flap because of a small area of necrosis. Trastuzumab for the remaining of one year and tamoxifen therapy are planned. The need for postoperative radiation therapy to the right chest wall and/or regional nodal basins was discussed at the Multidisciplinary Tumor Board, given her original presentation with a positive axillary node. There were divergent opinions regarding the need for XRT given the complete pathologic response. Ultimately, the patient decided not to receive postoperative radiation therapy.

## 2. Discussion

Neoadjuvant chemotherapy has become the standard of care for patients with locally advanced breast cancer and is currently considered a reasonable alternative to adjuvant chemotherapy for patients with large operable disease [1]. Pathologic complete response (pCR) after neoadjuvant chemotherapy has been consistently associated with improved outcomes [2–5]. The most common definition of pCR includes absence of invasive carcinoma in the breast and axillary nodes. Residual DCIS after NC does not portray worse prognosis compared to complete eradication of all disease [6, 7]. However, even if presence of residual DCIS does not affect long-term outcome, it has clinical implications regarding the surgical management of the patient and may at times lead to the need for more extensive resections, including the need for mastectomy despite excellent response to neoadjuvant chemotherapy.

To our knowledge, complete eradication of documented extensive intraductal component with neoadjuvant chemotherapy has not been reported if there are persistent malignant-appearing microcalcifications at the time of surgery. Whether the intraductal component of a tumor can be eradicated with neoadjuvant chemotherapy is controversial [8, 9]. Matsuo et al. evaluated the concordance in pathologic response to neoadjuvant chemotherapy between the invasive and the noninvasive components of primary breast carcinomas in 100 patients receiving neoadjuvant chemotherapy [8]. They found a strong correlation in pathologic complete response between the invasive and noninvasive components (*P* < 0.001). However, in that paper, they did not comment on the persistence or eradication of associated microcalcifications. On the other hand, Wu et al. [9] evaluated 25 patients with locally advanced breast cancer who received neoadjuvant chemotherapy with special attention to the proportion of intraductal component. They found that although neoadjuvant chemotherapy had a favorable effect on tumor reduction, its effectiveness varied depending on the predominance of the intraductal component. Cases with high proportion of intraductal component had lower response to neoadjuvant chemotherapy, and a large number of malignant cells remained in the mammary ducts of such cases. In addition, these residual cancer cells maintained proliferative activity. Based on their findings, they concluded that the intraductal component is poorly responsive to neoadjuvant chemotherapy.

Since the presence of malignant-appearing microcalcifications is usually a good surrogate for the presence of extensive intraductal component associated with invasive breast cancer, it is of interest to examine the fate of such microcalcifications following neoadjuvant chemotherapy. Several reports exist in the literature on the effect of neoadjuvant chemotherapy on microcalcifications [10–16]. The majority of these reports demonstrate no changes in the malignant-appearing microcalcifications with the administration of neoadjuvant chemotherapy. Junkermann and von Fournier observed no regression of microcalcifications following administration of neoadjuvant chemotherapy, even when the invasive tumor did show regression, hinting to the presence of noninvasive residual tumor [13]. Ferranti et al. [14] analyzed the morphology, number, and extent of the microcalcifications and assessed their value as reliable parameters of cancer response to primary chemotherapy. They found that increased visibility of the microcalcifications after chemotherapy was due to a reduction in both edema and lesion opacity. They concluded that microcalcifications are a useful parameter for diagnosis, but they alone are less important when evaluating response to primary chemotherapy. In some reports, microcalcifications are found to develop during neoadjuvant chemotherapy. Fadul et al. [16] described a patient diagnosed with stage III breast cancer and no microcalcifications prior to neoadjuvant treatment, who developed microcalcifications after treatment. These microcalcifications were histologically associated with both intraductal and invasive carcinomas. In contrary, some studies have reported a decrease in the number of microcalcifications with neoadjuvant chemotherapy or even complete disappearance. Adwani et al. [12] reported a case of complete resolution of all malignant-appearing microcalcifications after neoadjuvant chemotherapy, but Noguera Tajadura et al. found that microcalcifications evolve unpredictably and independently of tumor response to neoadjuvant chemotherapy [17]. Moskovic et al. [11] hypothesized that residual microcalcifications after neoadjuvant chemotherapy could be explained by the calcification of necrotic material remaining from the tumor or even fat necrosis or hematoma formation after biopsy. A study looking at the mammographic changes of 95 breast cancer patients after neoadjuvant chemotherapy found that patients with microcalcifications did not have complete response, and the prediction of pathologic outcome was not possible using mammograms [18]. Similarly, Segal et al. reported that microcalcifications may decrease in number, but rarely disappeared, and their persistence was usually associated with residual intraductal carcinoma [10].

One can question whether our observations represent true eradication of DCIS or merely the result of inadequate pathologic sampling of the tumor bed area. In support of a complete eradication of DCIS is the identification of several ducts that contained microcalcifications associated with high-grade DCIS on the original core biopsy specimen and similarly the identification of several ducts with microcalcifications that appeared normal on final pathology following neoadjuvant chemotherapy (Figures 5(a) and 5(b)).

Trastuzumab has been shown to be an effective anti-HER2 targeted therapy when used in the adjuvant setting for invasive breast cancer. Clearly, the addition of trastuzumab to chemotherapy in patients with HER-2-positive invasive carcinoma has been associated with considerable increase in pCR rates. It has been proposed that trastuzumab may downstage DCIS and possibly prevent transition from DCIS to invasive breast cancer [19]. Because DCIS is earlier in the carcinogenic pathway, it is more likely to depend on a single pathway rather than alternate escape pathways. Currently, new studies evaluating adjuvant and neoadjuvant trastuzumab and its effect on HER2-overexpressing DCIS are enrolling (NSABP B-43, MD Anderson DCIS neoadjuvant trial) [19].

We believe that this is the first report of documented complete eradication of the noninvasive component despite the persistence of malignant-appearing microcalcifications in a patient receiving neoadjuvant chemotherapy plus trastuzumab. Whether the addition of trastuzumab to chemotherapy contributed to this result is unknown at present.

Our observation, if reproduced by others, particularly in clinical trials comparing neoadjuvant chemotherapy versus neoadjuvant chemotherapy plus trastuzumab, may have clinical implications regarding surgical management of patients with HER-2 neu-positive breast cancer who have excellent clinical and radiologic response to neoadjuvant chemotherapy plus trastuzumab but have residual microcalcifications. In such cases, core biopsy confirmation of the true nature of residual microcalcifications may be needed before proceeding with more extensive surgical resection based on the extent of microcalcifications.
